# Clinical features of thymoma with and without myasthenia gravis

**DOI:** 10.12669/pjms.40.7.8698

**Published:** 2024-08

**Authors:** Yaxuan Wang, Guoyan Qi, Ying Yang, Hongxia Yang

**Affiliations:** 1 Yaxuan Wang, Department of Oncology, Hebei Medical University, Shijiazhuang 050017, Hebei, China; 2 Guoyan Qi, Department of Oncology, Hebei Medical University, Shijiazhuang 050017, Hebei, China. Hebei Provincial Key Laboratory of Myasthenia Gravis, Shijiazhuang 050030, Hebei, China; 3 Ying Yang, Center of Treatment of Myasthenia Gravis, People’s Hospital of Shijiazhuang Affiliated to Hebei Medical University, Shijiazhuang 050017, Hebei, China; 4 Hongxia Yang, Center of Treatment of Myasthenia Gravis, People’s Hospital of Shijiazhuang Affiliated to Hebei Medical University, Shijiazhuang 050017, Hebei, China

**Keywords:** Myasthenia gravis, Thymoma, Clinical, Pathology

## Abstract

**Objective:**

To explore the clinical features of thymoma with and without myasthenia gravis (MG).

**Methods:**

This was a retrospective study. Two hundred and thirty-three patients with mediastinal masses who were initially diagnosed in People’s Hospital of Shijiazhuang, China, between January 2014 and June 2022 and had complete clinical data and underwent surgical treatment at People’s Hospital of Shijiazhuang were retrospectively analyzed.

**Result:**

The age of patients with thymoma alone was significantly older than that of thymoma patients complicated with MG. The number of female patients was slightly more than males for both groups. Proportions of type A, AB, B1, B2, and B3 thymomas in Group-A were 0.77, 11.54, 11.51, 33.85, and 31.54%, respectively, and the proportions in Group-B were 9.68, 22.58, 12.90, 32.26, and 22.58%. The size of tumors in patients with thymoma alone was larger than that of patients with thymoma complicated with MG. The proportion of patients with tumor size of more than 10 cm in the thymoma alone group was significantly higher than that in the MG group. There were no relapses in patients with type A disease and relapses were noted in a few patients with type B1, B2 and B3 diseases. The same survival rates were reported for the two groups.

**Conclusion:**

MG rarely occurs in type A and type C diseases. The prognosis of thymoma with MG is similar to that of thymoma alone. The main causes of death may be myasthenia crisis in thymoma patients with MG and advanced tumor stage in patients with thymoma alone.

## INTRODUCTION

Thymoma is a rare tumor in the anterior superior mediastinum and arises from the thymic epithelium. It represents about 50% of anterior mediastinal tumors and less than 0.5% of the known tumors in humans.^1^ Thymoma in the mediastinum tends to invade the adjacent heart and great vessels, and often produces autoimmune cells and antibodies, leading to the development of autoimmune diseases such as MG. Epidemiological studies have found that about 30-50% of thymoma patients are complicated with MG.^2^,^3^ Complete surgical resection is currently the main treatment for thymoma, but there are still risks of relapse after surgical resection. Some studies^4^,^5^ mainly focused on the presence of MG and its impact on the prognosis of thymoma patients. Whether the early discovery of thymoma caused by MG is associated with a favorable prognosis and whether the development of myasthenic crisis will increase the number of deaths in thymoma patients with MG are still controversia.l,^6^,^7^ In the present study, the age, sex, Osserman classification, surgical methods, surgical staging, and thymus pathology of thymoma patients with and without MG who were initially treated in People’s Hospital of Shijiazhuang were reviewed and analyzed.

## METHODS

Two hundred and thirty-three patients with mediastinal masses who were initially diagnosed in People’s Hospital of Shijiazhuang between January 2014 and June 2022 and had complete clinical data and underwent surgical treatment at People’s Hospital of Shijiazhuang were retrospectively analyzed. Follow up of 161 cases, who diagnosed with thymoma by postoperative pathology were divided into two groups: Group-A with MG(n=130) and Group-B without MG(n=31). Patients were screened according to the inclusion and exclusion criteria.

### Ethical Approval:

The study was approved by the Institutional Ethics Committee of People’s Hospital of Shijiazhuang (No.: 94; Date: November 30, 2018), and written informed consent was obtained from all participants.

### Inclusion criteria:

- Patients who underwent preoperative chest CT examination and had definite postoperative pathological evidence of thymoma;
- Patients in Group-A underwent neostigmine test, repetitive nerve stimulation test, and acetylcholine receptor antibody (AchRAb) test, with a definite diagnosis of MG;
- Patients who had complete clinical and pathological data and follow-up data.

### Exclusion criteria:

- Patients who received preoperative radiotherapy, chemotherapy, and high-dose hormone therapy;
- Patients with malignant tumors in other parts of the body and patients with metastatic liver cancer.

### Diagnostic criteria for MG:

Typical manifestation of skeletal muscle fatigue, including ptosis, diplopia, limb weakness, difficulty chewing and swallowing, and dyspnea; positive in neostigmine test; positive in acetylcholine receptor antibody test; the amplitude of low-frequency fluctuation decreased by >10% in repetitive nerve stimulation in electromyography, with no increase in the amplitude of high-frequency fluctuation^6^. Diagnosis of MG was made if any two of the four criteria were met.

### Diagnostic criteria for thymoma:

Definite postoperative pathological evidence of thymoma. MG was classified according to the modified Osserman classification system: Type-I: ocular muscle only; Type-IIA: mild generalized; Type-IIB: moderate generalized; Type-III: acute severe; Type-IV: delayed severe; and Type-V: muscular atrophy. Among them, Types-IIA-V were generalized MG. No patients with Type-V disease were found in the present study.

The pathological classification was performed according to the latest WHO histological classification criteria in 2015, and MG was classified into types A, AB, B1, B2, B3, micronodular thymoma with lymphoid stroma, metaplastic thymoma and other rare types (microthymoma, sclerosing thymoma, and lipofibroadenoma). Masaoka staging system was used for clinical staging of thymoma: *stage I:* no microscopic transcapsular invasion, with completely encapsulated tumor; *stage II:* microscopic transcapsular invasion, invasion into adipose tissue or surrounding pleura; *stage III*: invasion into neighboring organs, i.e., great vessels, pericardium, or lung; *stage IVa*: pleural or pericardial dissemination; *stage IVb*: lymphogenous or hematogenous metastasis.

### Statistical Analysis:

SPSS26.0 statistical software was used for data analysis. Independent sample t-test was used for inter-group comparison, and analysis of variance was used for multigroup comparison. Enumeration data were compared using the χ^2^ test. Survival time was calculated as the time from the day of surgery to death or the last follow-up, and the Kaplan Meier survival curve was used to compare the survivals between the two groups. P-values <0.05 were considered statistically significant.

## RESULTS

Among the 233 patients with mediastinal masses, two hundred patients who were postoperatively diagnosed with thymoma were included and 26 patients with thymic cyst, three with thymic carcinoma, two with thymic abscess, one with teratoma and one with nodular hyperplasia were excluded. Among the patients included, five patients who received preoperative chemoradiotherapy, 34 patients who received hormone pulse therapy. Eligible patients were divided into Group-A (thymoma with MG, n=130) and Group-B (thymoma without MG, n=31). Clinical data collected from these patients were shown in Table-I. In Group-A, two patients received double filtration plasmapheresis prior to surgery, 80 received anticholinesterase treatment, and four received pulse therapy with human immunoglobulin. Mediastinum was evaluated by contrast-enhanced computed tomography before thymectomy. Surgical methods included extended thymectomy via the transsternal approach (n= 8) or video-assisted thoracoscopic surgery (VATS, n= 153). In 130 thymoma patients with MG, almost all of them were admitted with symptoms of MG. Among 31 patients with thymoma alone, 19 were found by chance in physical examination, and eight were found because of cough and chest discomfort.

**Table-I:** Clinical features of 161 patients with primary thymoma.

	A	B	p
n	130	31	
Female/male	68/62	17/14	0.8
Age of onset (years)	50.51±11.69	55.48±12.39	0.037
≤40	32(24.61%)	4(12.90%)	0.160
41-50	25(19.23)	9(29.03%)	0.206
51-60	44(33.85%)	6(19.35%)	0.117
≥61	29(22.31%)	12(38.71%)	0.045
***Tumor size***			
≤3cm	59(45.38%)	13(41.94%)	0.839
3.1-5.0	43(33.08%)	6(19.35%)	0.161
5.1-10.0	27(20.77%)	10(32.26%)	0.172
> 10	1(0.77%)	2(6.45%)	0.032
***WHO classification***			
A	1(0.77%)	3(9.68%)	0.026
AB	15(11.54%)	7(22.58%)	0.108
B1	15(11.54%)	4(12.90%)	0.832
B2	44(33.85%)	10(32.26%)	0.866
B3	41(31.54%)	7(22.58%)	0.327
Micronodular thymoma with lymphoid stroma	3(2.31%)	0(0%)	
Microthymoma	11(8.46%)	0(0%)	
***Masaoka stage***			
I	25(27.69%)	11(35.48%)	0.391
II	67(51.54%)	15(48.39%)	0.752
III	24(18.46%)	3(9.68%)	0.239
IV	3(2.31%)	2(6.45%)	0.216
***Surgical methods***			
Transsternal approach	7	1	
VATS	123	30	

The number of female patients was slightly higher than that of males in both groups. Patients with MG were significantly younger than those without MG (50.51 ± 11.69 vs 55.48 ± 12.39, p=0.037). Among patients ≥61 years old, the proportion of patients with MG was significantly lower than that of patients without MG, and compared with patients with thymoma alone, the tumor size in thymoma patients with MG was significantly smaller, mainly ≤5 cm in diameter; the proportion of Type-A thymoma was lower; and there were three patients with micronodular thymoma with lymphoid stroma and 11 with microthymoma, which were not found in patients with thymoma alone. No significant difference in the distribution of the Masaoka stage of thymoma was noted between the two group. Table-I.

The Masaoka staging and pathological classification of thymoma patients with myasthenia gravis were shown in Table-II. B1 type thymoma and microthymoma were mainly Masaoka stage I, while other pathological types are mostly Masaokao stage II, indicated that most thymomas were limited to intrathymic growth. The distribution of myasthenia gravis subtypes and pathological subtypes in this type of patient are shown in Table-III. Except for micro nodular thymoma with lymphoid stroma, which was mainly ophthalmic type, the other pathological subtypes were more common in systemic myasthenia gravis, mainly Osserman IIB type. Table-III.

**Table-II:** Masaoka stage and WHO pathological type of thymoma patients with MG.

Histological type	Masaoka stage				Total

	I	II	III	IV	
A	0	1	0	0	1
AB	5	9	1	0	15
B1	8	4	2	1	15
B2	7	29	7	1	44
B3	4	22	14	1	41
Micronodular thymoma with lymphoid stroma	1	2	0	0	3
Microthymoma	11	0	0	0	11
Grand total	36	67	24	3	130

**Table-III:** Classification of MG and pathological type of thymoma.

Histological type	Myasthenia gravis clinical staging					Total

	I	IIA	IIB	III	IV	
A	0	0	1	0	0	1
AB	4	3	7	1	0	15
B1	2	1	9	2	1	15
B2	8	7	20	7	2	44
B3	9	5	21	6	0	41
Micronodular thymoma with lymphoid stroma	2	1	0	0	0	3
Microthymoma	0	1	6	3	1	11
Grand total	25	18	64	19	4	130

Almost all thymoma patients with MG were ACHR-Ab positive. Only one microthymoma patient with MG was AchR-Ab negative. There was no significant change in the antibody titer levels of thymoma patients with ACHR-Ab positive before and after surgery, as shown in Table IV. No significant correlation was found between the overall antibody titer and the stage and pathological type of thymoma. Table-V, Table-VI. The level of acetylcholine antibody titer was significantly lower in ocular MG than that in generalized MG (p=0.042).

**Table-IV:** Comparison of postoperative ACHR-Ab levels in thymoma patients with MG.

AchRAb before thymoma surgery (nmol/L)	AchRAb after thymoma surgery (nmol/L)	p
9.79±4.18	9.57±4.24	0.166

**Table-V:** Relationship between AchRAb level and Masaoka stages in thymoma patients with MG.

	Thymoma Masaoka stage				p

	I	II	III	IV	
AchRAb(nmol/L)	9.41±4.64	10.16±3.96	9.71±3.43	7.14±4.05	0.547

**Table-VI:** Relationship between AchRAb level and pathological classification in thymoma patients with myasthenia gravis.

	WHO pathology type							p

	A	AB	B1	B2	B3	Micronodular thymoma with lymphoid stroma	Microthymoma	
AchRAb((nmol/L)	--	9.19±2.32	9.49±3.98	10.67±3.91	10.03±3.94	7.05±3.61	6.94±6.14	0.307

No postoperative adjuvant treatment was given to patients with microthymoma alone, type A, or stage-I diseases and thymoma patients complicated with MG received postoperative treatment with Buzhong Yiqi Decoction. Most patients with stage II and above and type AB or B diseases received radiotherapy, and some received systemic chemotherapy. Some patients with well-defined symptoms of MG after surgery received human immunoglobulin pulse therapy, double filtration plasmapheresis, glucocorticoid pulse therapy, and immunosuppressive therapy.

**Fig.1:**
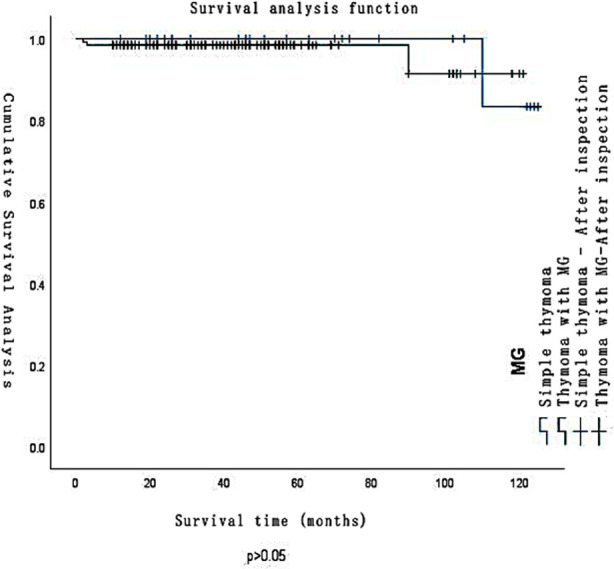
Survival curves for the two groups.

No perioperative deaths were reported among all patients. By the end of the follow-up, there was one death and one relapse in the thymoma-alone group. The pathological classification was type B2 and the Masaoka stage was stage-IV. There were three deaths and three local metastasis and relapses in the MG group, of which two were pathologically classified as B3 with Masaoka stage-III and IV, respectively, and one was type B1 with Masaoka stage I. No significant difference in survival rate (p=0.634). Fig.1 and relapse rate (p=0.775) was found between the two groups.

## DISCUSSION

In the present study, the proportion of Type-A thymoma in the MG group was significantly lower than that in the thymoma alone group. In most studies,^8^,^9^ the incidences of MG in thymoma patients were 0-14% for type A, 6-42% for Type-AB, 7-50% for type B1, 24-71% for Type-B2, and 25-65% for type B3. Thymoma complicated with MG can be any type of A, AB, and B1-3, with the highest incidence of Type-B2. The pathological results of thymoma in 130 patients in the present study also showed that Type-B2 was the most common one, which was consistent with the findings reported in the above studies.

Thymic epithelial tumors originate from the thymus and include thymic carcinoma and thymoma. Although thymoma is rare, accounting for about 0.2-1.5% of all malignant tumors,^10^ it is indeed the most common primary tumor in the anterior mediastinum and accounts for about 50% of all these primary tumors.^11^ Thymoma often induces the production of autoimmune T cells and antibodies and consequently leads to the development of autoimmune diseases, including MG. MG is an autoimmune disease. Previous studies have shown that MG is closely related to thymus, and MG occurs in 15-60% of patients with thymoma. In our study, at least 80.7% of thymoma patients were complicated with MG mainly because MG patients from all over the country were referred to our hospital for MG treatment and diagnosed as thymoma by CT. This inevitably resulted in a high proportion of thymoma patients complicated with MG in our data set. Due to the presence of MG, some differences might exist between patients with and without MG.Clinical features of thymoma were investigated and compared between patients with and without MG in the present study. Patients with thymoma underwent chest CT and MG-related tests at their first visit, and those with mediastinal tumors received surgical treatment. 80.7% of these patients were complicated with MG, and all patients were positive in the AchRAb test, except for one patient with microthymoma. Martinago et al.^12^ found in 2009 that MG with positive serum AChRAb was associated with thymoma or thymic hyperplasia. In undiagnosed patients with anterior mediastinal tumors, positive AchRAb almost certainly indicates a thymoma. AchRAb is not associated with other mediastinal tumors except for thymoma.

The production of anti-acetylcholine receptor antibodies is highly correlated with abnormal thymic microenvironment in thymoma. Shuey NH.^13^ found in 2008 that thymoma played an important role in triggering the autoimmune response to acetylcholine receptors. Anti-acetylcholine receptor antibodies were detected in thymoma patients complicated with MG. Abnormal thymic microenvironment can be removed by thymosectomy and the production of antibodies and autoreactive T cells is blocked in the thymus, resulting in a significant decrease in serum AChR antibodies.^14^ In the present study, however, no significant decrease was found in the level of anti-acetylcholine receptor antibodies one week after surgery compared with that before surgery. In the MG group of this study, there were 11 patients (8.46%) with microthymoma and three with micronodular thymoma with lymphoid stroma, both were not found in patients with thymoma alone. Microthymoma is a nest-like and agglomerated epithelial mass formed by the proliferation and aggregation of thymic epithelial cells in the thymus. Most microthymoma is multifocally distributed in the cortex, medulla or junctional areas. This thymoma cannot be detected in the radiological examination but can be found in the pathological diagnosis. In the present study, two patients with microthymoma were complicated with thymic cysts, and nine patients with MG were diagnosed after thymectomy. Cheuk et al.^15^ found microthymoma in the paraneoplastic thymus in three MG patients, which may represent the earliest stage of thymoma development. Its relationship with MG needs to be further confirmed in more patients. It has been reported that micronodular thymoma with lymphoid stroma can be complicated with type A or B2 thymoma.^16^

Similar histological and immunohistochemical phenotypes were found in the tumor epithelium of type A thymoma and mixed type of thymoma, i.e., micronodular thymoma with lymphoid stroma. It was speculated that type A thymoma and micronodular thymoma with lymphoid stroma may share common histogenesis. Although the proportion of concurrent MG is theoretically low, all three patients with micronodular thymoma with lymphoid stroma were complicated with MG, which may be explained by the establishment of the MG diagnosis and treatment center in our hospital in 2013. The proportion of patients with thymoma alone was significantly lower than that of thymoma patients complicated with MG.

Consistent with the findings reported in other studies,^17^ the onset ages of MG patients with thymoma in the present study tended to be younger than those of patients with thymoma alone, especially in the population over 60 years old with a significant difference. The number of female patients was slightly higher than that of males in both groups, with no significant sex difference in the overall population, which was consistent with the findings from previous studies.^18^

According to the clinical manifestations of patients, MG was divided into types I, IIA, IIB, III and IV using the Osserman classification system. Generalized MG was more common and accounted for 87.88% of thymoma patients complicated with MG, and 49.23% of generalized MG were type IIB diseases. Furthermore, type IIB MG was the most common disease in this Group-And accounted for over 40% of all pathological types of thymoma.

Moreover, patients with thymoma alone are often found by chance during physical examinations because of its relatively inert nature and slow growth, or discovered by the symptoms of compression caused by the large size of the tumor. With the popularization of chest CT in physical examinations over recent years, thymoma can be detected early and the proportion of early thymoma increases. Whether the prognosis of thymoma is affected by the presence of MG remains controversial. Some researchers suggested that MG is a prognostic factor for adverse outcomes for patients who undergo thymomatectomy because a myasthenic crisis may occur in MG, which leads to death from respiratory failure. While other authors believe that MG is a positive prognostic factor^19^, possibly owing to the early diagnosis of thymoma.

In the present study, the postoperative survival rates of thymoma patients complicated with MG and patients with thymoma alone were high, and the possible reasons included that patients who were not eligible for complete resection or patients who were treated with chemoradiotherapy before surgery were excluded from the present study. Therefore, the Masaoka stage of thymoma in this study was early. These results were consistent with the conclusion from most previous studies,^20^ i.e., complete tumor resection is the most important factor affecting the survival rate of thymoma.

### Limitations of this study:.

This was a retrospective descriptive study, with limited clinical data available and limited persuasive conclusions. Further intervention trials are needed in the future to confirm these results.

## CONCLUSIONS

MG rarely occurs in type A thymoma. There no significant difference in the survival rate between thymoma with MG and thymoma without MG. The main cause of death may be a myasthenic crisis for thymoma patients with MG and advanced tumor for thymoma patients without MG. Although thymoma is relatively inert, it is still invasive. Once diagnosed, patients with thymoma should undergo surgical treatment as soon as possible.

### Authors’ Contributions:

**YW,** **GQ:** Carried out the studies, data collection, drafted the manuscript, are responsible and accountable for the accuracy and integrity of the work.

**YY:** Performed the statistical analysis and participated in its design.

**HY:** Participated in acquisition, analysis, or interpretation of data and drafting the manuscript.

All authors read and approved the final manuscript.

## References

[1] Yu L, Ke J, Du X, Yu Z, Gao D (2019). Genetic characterization of thymoma. Sci Rep.

[2] Zhuang J, Guan M, Liu M, Liu Y, Yang S, Hu Z (2021). Immune-Related Molecular Profiling of Thymoma with Myasthenia Gravis. Front Genet.

[3] Lee MC, Hsiao TH, Chuang HN, Lee LW, Chi PL, Tsai HM (2020). Molecular profiling of thymoma with myasthenia gravis:Risk factors of developing myasthenia gravis in thymoma patients. Lung Cancer.

[4] Menon D, Katzberg H, Barnett C, Pal P, Bezjak A, Keshavjee S (2021). Thymoma pathology and myasthenia gravis outcomes. Muscle Nerve.

[5] Payet CA, You A, Fayet OM, Dragin N, Berrih-Aknin S, Le Panse R (2022). Myasthenia Gravis:An Acquired Interferonopathy?. Cells.

[6] Kondo K, Monden Y (2005). Thymoma and myasthenia gravis:a clinical study of 1,089 patients from Japan. Ann Thorac Surg.

[7] Lin X, Qi G (2022). Observation on the efficacy of different methylprednisolone regimens in the treatment of myasthenia gravis. Pak J Med Sci.

[8] Comacchio GM, Benetti B, Di Liso E, Menichetti A, Guarneri V, Rea F (2022). Induction chemotherapy with carboplatin and paclitaxel for thymoma in acute respiratory distress due to myasthenia gravis:a case report. Ann Palliat Med.

[9] Marx A, Belharazem D, Lee DH, Popovic ZV, Reißfelder C, Schalke B (2021). Molecular pathology of thymomas:implications for diagnosis and therapy. Virchows Arch.

[10] Willner J, Zhou F, Moreira AL (2022). Diagnostic Challenges in the Cytology of Thymic Epithelial Neoplasms. Cancers (Basel).

[11] Zhang Y, Lin D, Aramini B, Yang F, Chen X, Wang X (2023). Thymoma and Thymic Carcinoma:Surgical Resection and Multidisciplinary Treatment. Cancers (Basel).

[12] Martignago S, Fanin M, Albertini E, Pegoraro E, Angelini C (2009). Muscle histopathology in myasthenia gravis with antibodies against MuSK and AChR. Neuropathol Appl Neurobiol.

[13] Shuey NH (2022). Ocular myasthenia gravis:a review and practical guide for clinicians. Clin Exp Optom.

[14] Chen J, Wu X, Liu Y, Zhang W (2023). Changes and significance of serum AchR-Ab and CAE-Ab in patients with thymoma after thoracoscopic surgery. Biotechnol Genet Eng Rev.

[15] Cheuk W, Tsang WY, Chan JK (2005). Microthymoma:definition of the entity and distinction from nodular hyperplasia of the thymic epithelium (so-called microscopic thymoma). Am J Surg Pathol.

[16] Tsuchiya T, Sano A, Kawashima M (2021). Micronodular Thymoma with Lymphoid Stroma:A Case Report. Tokai J Exp Clin Med.

[17] Miura K, Doi T, Tanaka Y, Hokka D, Jimbo N, Itoh T (2022). Effect of myasthenia gravis on the surgical outcomes of patients with thymoma. Asian Cardiovasc Thorac Ann.

[18] Tang M, Shao Y, Dong J, Gao X, Wei S, Ma J (2023). Risk factors for postoperative myasthenia gravis in patients with thymoma without myasthenia gravis:A systematic review and meta-analysis. Front Oncol.

[19] Zhang Y, Yu L, Ke J (2022). Pathological Features and Prognosis of Thymoma with or Without Myasthenia Gravis. Front Surg.

[20] Kas J, Bogyo L, Feher C, Ghimessy A, Gieszer B, Karsko L (2022). Unilateral video-assisted thoracoscopic thymoma resection –Indications, early and mid-term results. Magy Seb.

